# Screening of vitamin D and calcium concentrations in neonates of mothers at high risk of vitamin D deficiency

**DOI:** 10.1186/s12887-020-02204-8

**Published:** 2020-07-04

**Authors:** Sheikh Arif M. Kozgar, Paul Chay, Craig F. Munns

**Affiliations:** 1 Department of Paediatrics, Latrobe Regional Hospital, Traralgon, Victoria Australia; 2 Monash University, School of Rural Health, Traralgon, Victoria Australia; 3 Department of Paediatrics, Liverpool Hospital, Liverpool, NSW Australia; 4 University of NSW, Faculty of Medicine, School of Women’s and Children’s Health, Sydney, Australia; 5 Department of Paediatric Endocrinology, The Children’s Hospital at Westmead, Westmead, NSW Australia; 6 Department of Paediatrics and Child Health, University of Sydney, School of Medicine, Sydney, Australia

**Keywords:** Vitamin D, Calcium, Neonates, High risk, Prevalence, Concentration or levels

## Abstract

**Objective:**

The aim of this study was to determine, retrospectively, the serum 25OHD and calcium concentrations of screened neonates of mothers at high risk of 25OHD deficiency and examine whether their measurement contributes to the management of these neonates.

**Methods:**

Serum 25OHD and calcium concentrations from 600 samples of umbilical cord blood or venous blood collected from neonates over a 12-month period were analysed.

**Results:**

There was a high prevalence of vitamin D insufficiency (27.6%, 30–50 nmol/L) and deficiency (21.3%, < 30 nmol/L) in neonates from high-risk maternal groups. There was a statistically positive but weak correlation (ρ = 0.22, *P* < 0.0001) between 25OHD and serum calcium. Only 7 neonates out of 569 (1.2%) had calcium concentrations in the hypocalcaemic range; however, a significant number (47.6%) were reported to be in the hypercalcaemic range. Nearly all of these were venous samples collected in first 24 h after birth.

**Conclusion:**

Vitamin D deficiency is prevalent in neonates of high-risk mothers but the risk of hypocalcaemia due to vitamin D deficiency at birth is low. Screening neonates entails blood testing which can cause distress to neonates and their parents, substantial imposition on staff and financial burden on the health care system. Vitamin D supplementation of these neonates from birth without routine screening appears more reasonable. Also, the data from this study suggest that the paediatric reference range for corrected calcium concentrations in neonates should be re-evaluated.

## Introduction

Vitamin D (25 hydroxy-vitamin D (25OHD)) deficiency is a global health problem and together with poor calcium intake is responsible for nutritional rickets and osteomalacia. When severe, it leads to fractures and skeletal deformities in growing infants and children as well as asymptomatic and symptomatic hypocalcaemia in the form of cardiomyopathy, tetany and seizures [1–4]. Although vitamin D is primarily required to maintain serum calcium homeostasis, there is increasing evidence that it may play a role in many other metabolic and physiological processes apart from maintaining bone health [5, 6]. Vitamin D deficiency is especially prevalent during pregnancy in women with dark skin pigmentation and/or reduced ultraviolet radiation exposure due to ethno-cultural factors such modest/concealed clothing, application of sunscreen or less outdoor activity due to chronic illness and obesity [7–11]. The re-emergence of nutritional rickets in countries like Australia is not surprising due to increased immigration and diversity of ethnic groups, and thus a high proportion of the population is in the high-risk category of vitamin D and calcium deficiency [3, 12, 13].

The risk of nutritional rickets is greatest when vitamin D deficiency and dietary calcium deficiency are combined. If a child is deficient in only vitamin D or calcium, adequate bone mineralisation can still be sustained [14]. An exception to this is neonates and infants, who are growing rapidly and need both adequate vitamin D and calcium intake for bone mineralisation [4, 14, 15]. Neonates of vitamin D-deficient mothers or those at risk of vitamin D deficiency can exhaust compensatory mechanisms quickly and become hypocalcaemic. The parathyroid hormone stimulates osteoclasts to increase bone resorption to maintain normocalcaemia and impaired renal phosphate absorption and low phosphate levels leading to nutritional rickets and osteomalacia [16, 17].

The management of neonates with maternal vitamin D deficiency or mothers at risk of vitamin D deficiency varies across regions in Australia. In some units, neonates are routinely started on cholecalciferol 400 IU daily, in others they are screened and/or tested, while in many others no screening or treatment protocol exists [18–20]. The policy in the paediatric unit at Liverpool Hospital (Sydney, Australia) [21] was to screen neonates for vitamin D deficiency by measuring their 25OHD concentrations or levels after birth if their mothers had 25OHD < 25 nmol/L detected during pregnancy or unknown 25OHD concentrations and risk factors for vitamin D deficiency (Table 1).

**Table 1:** Screening criteria for identifying mothers of neonates at high risk for vitamin D deficiency at Liverpool Hospital

1. 25OHD < 25 nmol/L **or**	
2. Unknown Vitamin D levels and risk factors	
**a**. Dark Skin	
**b**. History of poor sun exposure	
**c**. Veiled	
**d**. Chronic illnesses like inflammatory bowel disease, renal or liver disease	
**e**. Obesity	

Mothers at high risk of vitamin D deficiency were identified by midwives or nursing staff at the time of admission and neonatal cord blood samples obtained at birth. If that opportunity was missed, high risk neonates were themselves tested in the post-natal ward and then managed as per the 2006 Australia and New Zealand consensus statement guidelines [7].

### Aim of study

The objective of this study was to determine, retrospectively, the prevalence of vitamin D deficiency and hypocalcaemia in a cohort of ‘high risk neonates’, so as to examine contribution of measurements to the current screening protocol of these neonates.

## Methods

This single-centre retrospective study was conducted at Liverpool Hospital in western Sydney and was approved by the South Western Sydney Local Health District Human Research Ethics Committee. The population in this area is quite diverse, with 37% born overseas in a non-English speaking country [22]. Babies born at Liverpool Hospital between January and December 2015 and identified as high risk who had cord blood or venous blood tested for 25OHD and calcium concentrations within the first week of life were included. The levels were obtained from the hospital laboratory records, and the neonatal medical record was used to determine the babies’ gestational age, sex and birth weight. 25 hydroxy-vitamin D and calcium concentrations were measured by automated immunoassay. 25OHD was assayed on the DiaSorin Liaison XL analyser. The laboratory participated in the Vitamin D External Quality Assurance Survey (DEQAS) for international standardization of 25OHD assay. Calcium and albumin were analysed on the Roche Cobas 702 analyser.

**Fig. 1:**
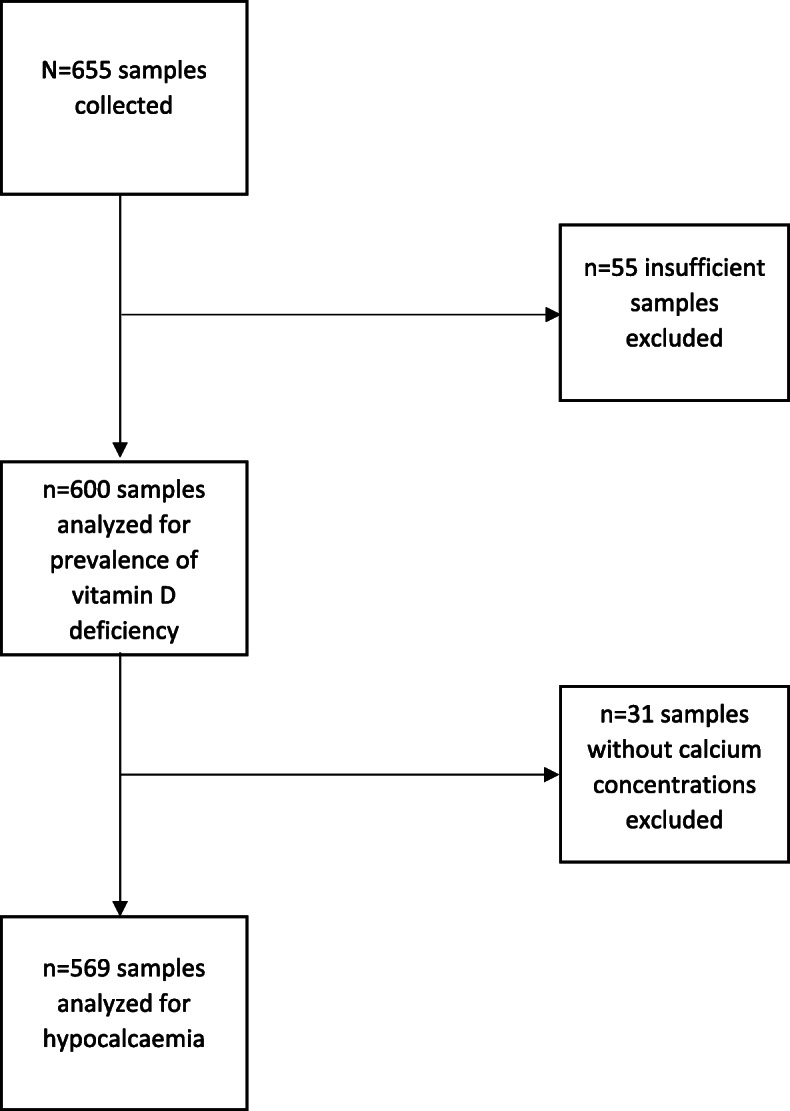
Flow chart illustrating number of samples analysed

A total of 655 samples were collected over a 12-month period, of which 55 were reported as insufficient and were excluded from the analysis. Of the remaining 600 samples, 25OHD concentrations were reported for all while both the corrected calcium concentrations and 25OHD were available for 569 samples (Fig. 1).

The corrected calcium concentrations were reported from the laboratory based on reference intervals from the Clinical Guide to Laboratory Tests [23] as follows: Cord blood sample ref. range 2.32–2.99 mmol/L and Venous blood sample ref. range 0–1 day 2.25–2.65 mmol/L; > 1–2 days 1.75–3.00 mmol/L and >  2–7 days 2.25–2.73 mmol/L.

### Statistical analyses

Clinical and patient characteristics were described by frequencies and percentages for categorical variables, while for continuous variables, median, mean (standard deviation) or range was used. Association between 25OHD and other variables was tested by chi-square test, t-tests, Pearson’s product-moment correlation and Spearman’s rank-order correlation. T-tests were used for gender (2 groups, male and female). For birth weight, gestational age and corrected calcium, cross-tabulation and chi-square tests were done with both variables categorised and correlation coefficient and scatter plots for both variables continuous. Differences were considered statistically significant when p- values were less than 0.05. There was no adjustment made for multiple statistical comparisons. SAS 9 statistical software was used for analysis and the reference interval of corrected calcium was calculated with statistics program Analyse It.

## Results

The gender distribution was nearly equal in the sample of 600. Neonates were predominantly born at term gestation (≥ 37 weeks) with a mean age of 38.6 weeks and were predominantly of normal birth weight (≥ 2500 g) with mean birth weight of 3212 g (Tables 2 and 3). Cord blood made up 20.3% of the samples while the rest were venous samples. Most neonates had a cord blood or venous blood test done on the first day of life (81.1%) and nearly all samples were collected within 4 days of birth.

**Table 2:** Variables

Variables		Frequency	Percent
**Gender**	**Female**	285	47.50
	**Male**	315	52.50
**Term**	**Pre-term (< 37 weeks)**	71	11.83
	**Term (≥ 37 weeks)**	529	88.17
**Birth Weight**	**Low (< 2500)**	67	11.17
	**Normal (≥ 2500)**	533	88.83
**Sample type**	**Cord blood**	122	20.61
	**Venous**	478	79.39
**Test age**	**0 to 1 day**	487	81.17
	**> 1 to 2 days**	50	8.33
	**> 2 to 7 days**	63	10.50

**Table 3:** Statistical analysis of variables

Variables	Number (n)	Mean	Standard Deviation	Median	Lower Quartile	Upper Quartile	Minimum	Maximum
**Gestational age (weeks)**	600	38.6	2.4	39.0	38.0	40.0	26.0	42.0
**Birth Weight (grams)**	600	3212	654	3270	2900	3620	520	4760
**25OHD (nmol/l)**	600	56	30	51	32	76	9	198
**Corrected Calcium (mmol/l)**	569	2.67	0.18	2.68	2.54	2.78	1.90	3.28

**Fig. 2:**
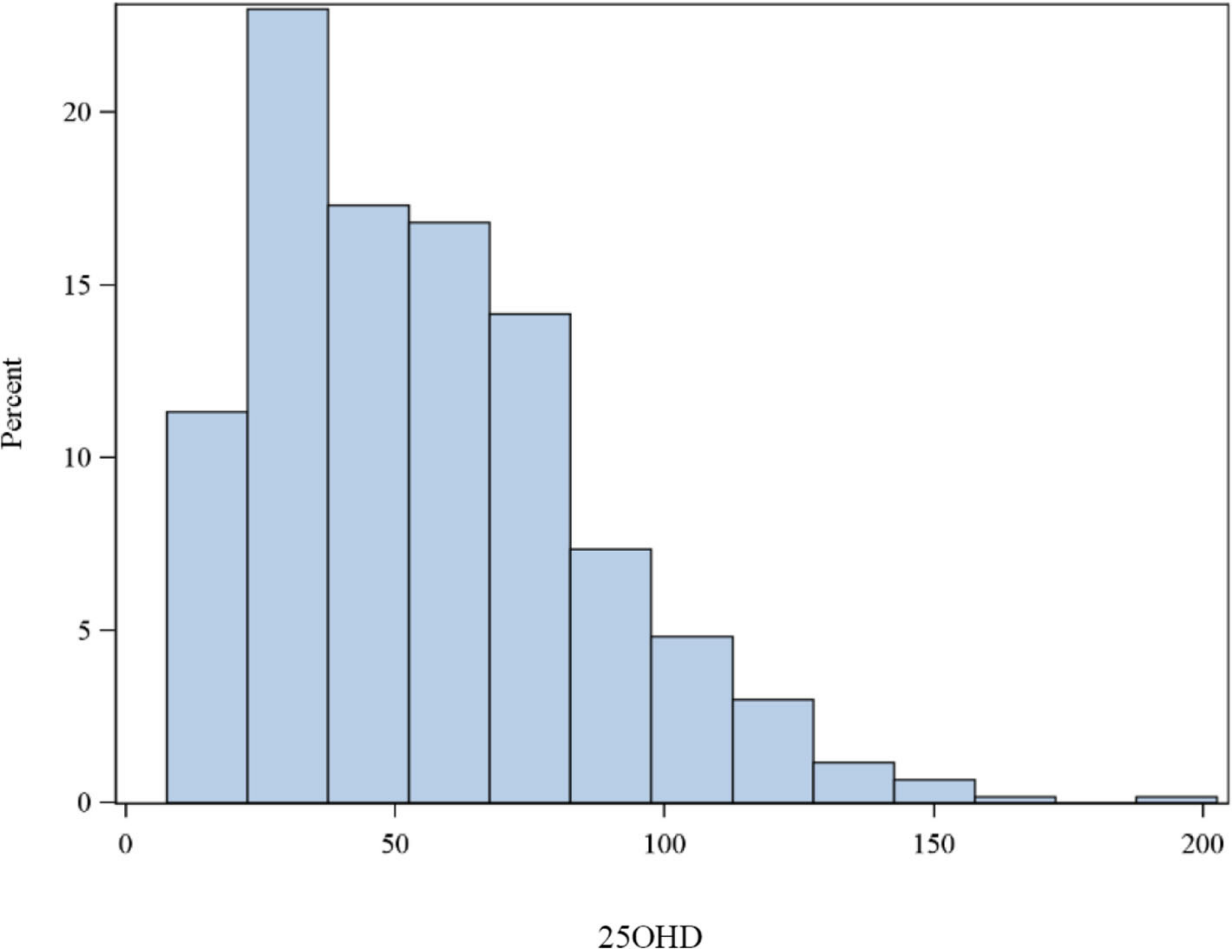
Distribution of serum 25 hydroxy-vitamin D (nmol/L)

There was little or no evidence of association between neonatal 25OHD concentrations and the birth variables of gender, gestational age or birth weight. According to the classification of vitamin D deficiency from the International Global consensus guidelines 2016 [24] vitamin D levels were sufficient (25OHD > 50 nmol/L) in 51% of neonates, insufficient (25OHD 30–50 nmol/L) in 27.6% and deficient (25OHD < 30 nmol/L) in 21.3%. This indicated a high prevalence of vitamin D insufficiency and deficiency in high-risk maternal groups screened for vitamin D levels. (Fig. 2).

**Fig. 3:**
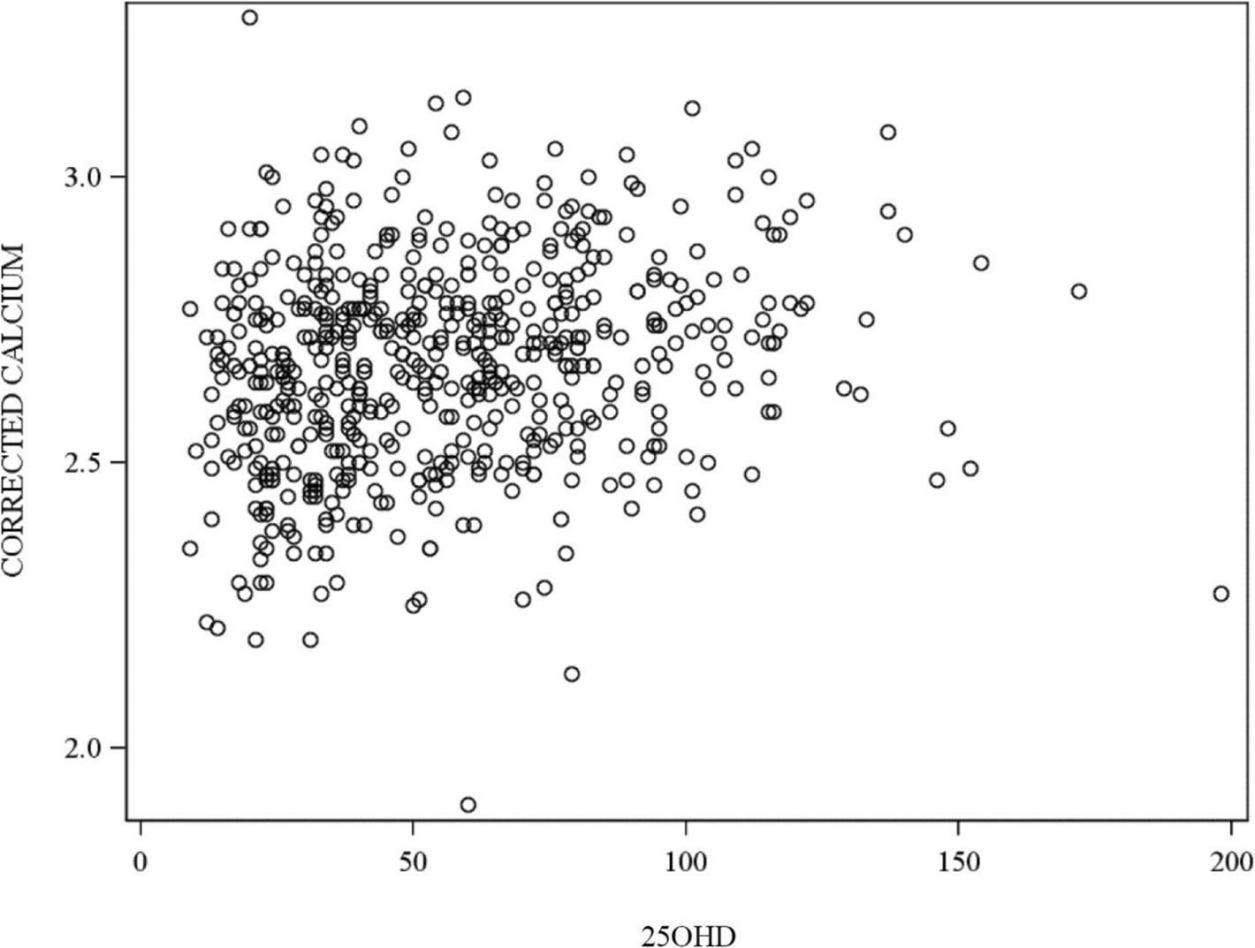
Scatter plot showing relationship between serum 25 hydroxy-vitamin D (nmol/L) and corrected calcium (mmol/L)

There was overall statistically positive correlation between vitamin D and corrected calcium concentrations (*P* < 0.0001). However, the strength of the correlation was weak (ρ = 0.22) (Fig. 3).

The corrected calcium concentrations were reported within the normal range in about half of the 569 samples available while levels were in the hypercalcaemic range in 47.6%. Nearly all the hypercalcaemic values were those of venous samples collected on first day of life. Only three venous samples collected after day 1 were in the hypercalcaemic range while none of the cord blood samples collected at birth were hypercalcaemic (Table 4).

**Table 4:** Corrected calcium levels (mmol/L)

***CORRECTED CALCIUM***					
***Sample***	***Test age***	***Calcium level***	***No.***	***Minimum***	***Maximum***
*Venous*	*0 to 1 day*	*Hypocalcaemia*	*2*	*1.90*	*2.19*
		*Normal*	*82*	*2.26*	*2.65*
		*Hypercalcaemia*	*268*	*2.66*	*3.28*
	*> 1 to 2 days*	*Normal*	*45*	*2.13*	*2.77*
	*> 2 to 7 days*	*Hypocalcaemia*	*2*	*2.19*	*2.22*
		*Normal*	*45*	*2.25*	*2.72*
		*Hypercalcaemia*	*3*	*2.75*	*3.08*
*Cord blood*	*0 to 1 day*	*Hypocalcaemia*	*3*	*2.21*	*2.27*
		*Normal*	*119*	*2.33*	*2.97*
		*Hypercalcaemia*	*0*	*–*	*–*

The incidence of hypocalcaemia was incredibly low (1.2%). Out of the seven hypocalcaemic neonates, three were preterm, one was low birth weight and three had sufficient 25OHD concentrations (Table 5).

**Table 5:** Characteristics of hypocalcaemic neonates and serum 25OHD levels

***Corrected calcium (mmol/L)***	***Gender***	***Gestational age (weeks)***	***Test age (days)***	***Birth weight (grams)***	***25OHD level (nmol/L)***	***Sample type***
*1.9*	*m*	*39.1*	*0.88*	*3180*	*60*	*Venous*
*2.19*	*m*	*39*	*0.8*	*3500*	*31*	*Venous*
*2.19*	*f*	*36*	*2.19*	*2532*	*21*	*Venous*
*2.21*	*m*	*35*	*0.13*	*2600*	*14*	*Cord blood*
*2.22*	*m*	*39*	*2.99*	*2780*	*12*	*Venous*
*2.26*	*f*	*39.5*	*0.04*	*3910*	*70*	*Cord blood*
*2.27*	*f*	*35*	*0.12*	*2190*	*198*	*Cord blood*

In this study, using the corrected calcium concentrations measured in venous blood in the first 24 h of life, we calculated a normal reference range of 2.38–3.04 mmol/L for corrected calcium. The upper limit of this calculated reference range is significantly higher than the standard reference range used in the laboratory at Liverpool Hospital (2.25–2.65 mmol/L).

## Discussion

This is the first study, to our knowledge, to critically examine the practice of screening neonates of high maternal risk for vitamin D deficiency. The absence of a correlation between birth variables and neonatal 25OHD concentrations from our data is consistent with other studies [25–27]. However, our study found a higher prevalence of vitamin D deficiency and insufficiency than previously reported by Bowyer et al. in Australia [8] and is comparable to prevalence recorded in mixed ethnic populations of other Western nations [28, 29]. The high prevalence of vitamin D deficiency in predominantly non-white regions like Africa and India is well known [30]; nonetheless, high prevalence of vitamin D deficiency is documented in regions at high latitude with a majority of fair skinned people and in other studies of mainly white ethnic populations [26, 31–33]. It seems then measuring 25OHD in high-risk neonates is unnecessary, given that an increased prevalence of vitamin D deficiency has been well established in these groups. One could argue that neonates may need screening to treat them according to the severity of their vitamin D deficiency to prevent complications. We found a very low incidence of hypocalcaemia and no relationship between severity of vitamin D deficiency and hypocalcaemia at birth as well as no reports of clinical seizures in those neonates. Also, there is evidence that even significantly low 25OHD concentrations in term neonates are readily corrected after birth with oral vitamin D supplementation as early as 6 weeks after treatment [34, 35]. Moreover, a systematic review by Mimouni et al. of randomised controlled trials involving vitamin D supplementation from birth to 23 months of age concluded no benefit of doses more than 400 IU for bone mineralisation. There was no effect on long-term outcomes with increased doses; rather, higher doses were potentially associated with adverse effects [36].

There are additional disadvantages of routine testing: cord blood samples are not available in the majority of cases and venepuncture causes undesirable effects of inflicting pain to babies and stress to parents [37]. It takes considerable staff time in organising for the tests, follow up of test results, communicating results to parents, arranging further follow up and thus significant financial costs to the health services [37, 38]. Besides, over half of the neonates were vitamin D sufficient on testing and were not supplemented. Nevertheless, they are at risk of developing vitamin D deficiency if they were exclusively breast fed or until sufficient feed volume is reached in formula fed infants [2, 7]. Hence, most international guidelines recommend oral supplementation with vitamin D for all infants [24, 39]. The major challenge to daily infant cholecalciferol supplementation remains poor adherence [40, 41] which is substantially improved with education and emphasis on cholecalciferol supplementation from health care providers or paediatricians in early post-partum period [42, 43].

We found an overall positive correlation between 25OHD and corrected calcium; however, the strength of the correlation was weak. This is in agreement with the study of Hillman et al. where they documented serial measurements of total calcium and 25OHD levels in term and premature neonates [44]. The correlation between neonatal vitamin D levels and neonatal hypocalcaemia at birth nevertheless is not clear in the literature [45]. Our study indicated a very low incidence of hypocalcaemia at birth, even with severe neonatal vitamin D deficiency. This is corroborated by case reports of symptomatic hypocalcaemia due to vitamin D deficiency usually presenting after first week of life [46–49]. Thus, testing for hypocalcaemia due to vitamin D deficiency early at birth is not reasonable.

Nearly half of the corrected calcium levels in our study were in the hypercalcaemic range and nearly all of them were venous samples in the first 24 h after birth. Although neonates have higher calcium levels at birth and cord blood calcium levels correlate well with maternal calcium levels [50], the calcium levels drop after birth over the first 12–48 h in neonates [51]. We postulate that the reason for the very high number of hypercalcaemic values may be due to the low upper limit of the reference interval used in the laboratory for venous samples collected in first 24 h [23]. We calculated the reference interval for corrected calcium of venous samples in first 24 h from our data and the upper limit was significantly higher. Many laboratories still use reference intervals for paediatric populations derived from old studies using obsolete equipment, adult populations or unwell children in hospital, all of which are inaccurate [52]. There are initiatives to establish more accurate reference intervals for paediatric populations [53–55] however further studies are required to establish correct reference intervals for corrected calcium in neonates.

The study was limited by the fact that we did not have full maternal data to pair mother–infant groups and compare maternal vitamin D status during pregnancy with neonatal 25OHD and calcium levels. A chemiluminescent immunoassay was used for 25OHD measurements rather than the gold standard liquid chromatography-tandem mass spectrometry. We did not have calcium levels for 31 out of 600 samples; however, this is unlikely to have influenced the results. Although no seizures were reported in the hypocalcaemic neonates in our study, we cannot rule out other symptoms of hypocalcaemia.

## Conclusion

Vitamin D deficiency is highly prevalent in our mixed-ethnicity population, and neonatal screening of vitamin D levels affirms what is largely known. Neonates must undergo an invasive procedure if cord blood is not available which causes pain to neonates, provokes anxiety in parents and stretches hospital resources. In addition, we found a very low incidence of hypocalcaemia in these healthy neonates with vitamin D deficiency at birth. It also appears that vitamin D deficiency is corrected relatively easily in neonates with supplementation. An alternative model of care of supplementing these babies with cholecalciferol without routine testing appears to offer better value of care.

We found an unusually high incidence of hypercalcaemia in neonates in the first 24 h of life likely due to unsubstantiated normative serum calcium range being used. We calculated a higher upper limit of reference range for corrected calcium. The data from this study suggests there is a need for ratification of reference ranges for corrected calcium levels in neonates.

## Data Availability

The datasets during and/or analysed during the current study available from the corresponding author on reasonable request.

## Abbreviations

25OHD: 25 hydroxy-vitamin D
IU: International Units
ref. range: Reference range
